# Effect of bulk material on the reliability and failure mode of narrow implants

**DOI:** 10.1111/eos.70021

**Published:** 2025-06-11

**Authors:** Ernesto B. Benalcázar‐Jalkh, Adolfo C. O. Lopes, Edmara T. P. Bergamo, Laura F. de Carvalho, Lukasz Witek, Paulo G. Coelho, Abbas Zahoui, Estevam A. Bonfante

**Affiliations:** ^1^ Department of Prosthodontics and Periodontology Bauru School of Dentistry University of Sao Paulo Bauru São Paulo Brazil; ^2^ Department of Prosthodontics NYU College of Dentistry New York New York USA; ^3^ Biomaterials and Regenerative Biology Division NYU College of Dentistry New York New York USA; ^4^ Department of Biomedical Engineering New York University Tandon School of Engineering Brooklyn New York USA; ^5^ Department of Oral and Maxillofacial Surgery NYU College of Dentistry New York New York USA; ^6^ Hansjörg Wyss Department of Plastic Surgery New York University Grossman School of Medicine New York New York USA; ^7^ DeWitt Daughtry Family Department of Surgery Division of Plastic Surgery University of Miami Miller School of Medicine Miami Florida USA; ^8^ Department of Biochemistry and Molecular Biology University of Miami Miller School of Medicine Miami Florida USA

**Keywords:** dental implants, fatigue, implant‐supported, titanium

## Abstract

The aim of the study was to assess the effect of bulk material on the reliability and failure modes of narrow‐diameter implants. Narrow implants (Ø3.5 × 10 mm ‐ 11° internal conical connection) were manufactured from three different bulk materials: commercially pure titanium grade‐IV (CP4), cold‐worked titanium (CW), and 4Titude (4Ti), and were evaluated under fatigue testing. Eighteen samples per group were tested under step‐stress accelerated life testing through 30° off‐axis load application in mild, moderate, and aggressive loading profiles. The number of cycles and load at failure were used to calculate use‐level probability curves and reliability for missions of 100,000 cycles up to 200 N, followed by fractographic analyses. Beta values suggested that damage accumulation dictated failures. Reliability analyses at 80, 120, and 150 N evidenced high reliability for narrow implants independent of bulk material. At 200 N, a decrease in reliability was observed for all groups (∼46%). Failure mode analysis depicted similar failures for all groups and comprised implant fracture, abutment fracture, and implant + abutment fractures. Narrow implants presented high reliability for physiologic masticatory forces in the anterior region. Characteristic strength, reliability, and failure modes were similar regardless of bulk material, suggesting that fatigue damage accumulation at thin wall implants dictated failure over bulk material strength.

## INTRODUCTION

Dental implants have been in widespread use for over five decades to support various types of prostheses, including single‐tooth replacements, multiple teeth, and full‐arch restorations. Although biological and mechanical complications are somewhat frequent, long‐term clinical trials have reported high survival rates for dental implants [1, 2, 3, 4, 5, 6, 7]. Among several factors required for the successful installation and restoration of dental implants, adequate bone availability is crucial to allow for the correct three‐dimensional positioning of the implanted device. Furthermore, sufficient bone availability is pivotal to prevent compromising the integrity of the osteotomy walls, exposing the implant threads, and hampering the predictability of the treatment [8, 9].

Agenesis, trauma, neoplasia resection, and high ridge atrophy after tooth extractions are commonly considered as challenging scenarios for ideal implant positioning [10]. Although bone grafting procedures have been proposed as an alternative to increase the alveolar ridge height/thickness and to facilitate precise implant placement, they are associated with notable drawbacks, including increased morbidity, extended healing periods, and elevated treatment expenses [11, 12]. Consequently, narrow diameter implants (< 3.75 mm) have emerged as a clinical solution for restoring regions with limited bone availability [12, 13, 14, 15].

While clinical assessments of narrow implant systems have indicated comparable survival rates when compared with standard diameter implants (Survival rates > 95%; marginal bone‐loss ∼2 mm after 4 years) [16, 17, 18], the thin walls of narrow implants, particularly those with internal connections, may lead to an increased risk of fracture. This represents a critical scenario, especially when subjected to repetitive physiologic loading, which may lead to the accumulation of damage over time [19, 20]. In fact, the mechanical assessment of reconstructions supported by implants of different diameters in vitro has demonstrated a higher survival rate for standard and wide implant systems than for narrow implants [21].

Considering the clear trend to implant dimension reduction noticed from early to current implant dentistry, implant bulk material selection has been reported to be of paramount importance due to mechanical performance and longevity concerns [22]. Since its first introduction in modern implantology, commercially pure titanium (CPT) grade II (ASTM F67) has been used traditionally as the bulk material of choice for the manufacture of dental implants [7]. However, the development of internal conical connections and the indication of reduced diameter dental implants have demanded the application of bulk materials with improved mechanical properties to reduce the risk of fracture of implants and components during oral function [23]. Therefore, CPT grade IV (ASTM F67) strained by cold working processing, and titanium alloys with other biocompatible metals such as vanadium (Ti‐6Al‐4 V; ASTM F136) have been used by manufacturers to improve the mechanical properties of conventional CPT grade II implants [24]. Further improvements involve the development of cold‐worked nanostructured grade IV CPT, which has been reported to present similar yield strength to that of Ti‐6Al‐4 V (*σ*_e _= 970 and 872 MPa, respectively) [25].

Although mechanical resistance is an important factor for bulk material selection, dental implants are subjected to the detrimental effect of cyclic loading during oral function, which has been shown to be critical in the lifetime evaluation of narrow implant devices [26]. While several in vitro studies have evaluated the fatigue performance and failure modes of narrow implants [20, 27‐30], most of them were conducted using two‐piece abutments, which have been reported to be the weakest component of these ensembles dictating failure modes [29, 31]. To overcome the strength limitation of two‐piece abutments, the use of monolithic abutments connected to internal conical implants has been suggested to improve the mechanical performance [20, 27, 28]. However, the use of bulkier abutments in narrow diameter implants may result in competing failures between the thin implant walls and the solid abutment [20].

Moreover, the evaluation of the mechanical integrity of implants retrieved after function has shown that commercially pure titanium implants exhibit more cracks and defects than Ti‐6Al‐4 V implants [32]. In addition to the correlation between implant material and the presence of defects, a concerning finding from the study by Shemtov‐Yona and Rittel [32] was that 62% of commercially pure titanium implants, which appeared intact but were removed due to biological complications, displayed cracks and defects of varying magnitudes. This suggests a significant potential for implant fractures due to damage accumulation from material fatigue. Thus, the use and development of materials with superior mechanical properties and greater resistance to fatigue‐induced damage are of great interest, particularly in narrow implants, to reduce the potential for long‐term catastrophic events under physiological repetitive loading. Therefore, the present study aimed to evaluate, through step‐stress accelerated life testing (SSALT), the probability of survival and the failure modes of narrow dental implants (3.5 mm) with identical macrogeometry manufactured with CPT grade IV, cold worked CPT grade IV, or 4Titude bar (Fort Wayne Metals), rehabilitated with monolithic abutments. The postulated null hypothesis of the present study was that no significant differences would be observed in the probability of survival and failure modes of narrow implants regardless of their bulk material.

## MATERIAL AND METHODS

### Sample preparation

Fifty‐four narrow implants (Ø 3.5 × 15 mm) with 11° tapered internal conical connections were evaluated in the present study. Implants were produced by the same manufacturer (SIN Implant System) with three different bulk materials to obtain three experimental groups (*n* = 18/group): (1) Commercially pure titanium grade IV (CP4); (2) cold‐worked grade IV titanium (CW), and (3) 4Titude titanium bars (4Ti; Fort Wayne Metals), a grade IV pure titanium with refined grain structure produced via severe plastic deformation. Implant bodies were machined using Swiss‐type automatic lathes with a sliding headstock. Thread machining was performed using a whirling tool equipped with 12 AlTiN‐coated carbide inserts. The process was executed in a single pass with a depth of cut of 0.25 mm, a feed rate of 0.9 mm, and a rotational speed of 3300 rpm. A plant‐based coolant (Plantocut 10SR, Fuchs) was used during the machining process. All implants were manufactured with the same macro design, a conical body with a large thread pitch, and deep, wide threads, and were rehabilitated with proprietary abutments for cemented restorations (Ø 3.3 mm x 5.5 mm x 6 mm, SIN implant system).

Using a dental surveyor (Delineador B2; Bio‐Art), all implants and their respective abutments were embedded in polymethylmethacrylate acrylic resin (Jet; Classico Artigos Odontologicos) into a silicon matrix with 15 mm diameter and 20 mm height. The implant platform was placed at the level of the surface of the potting acrylic resin. Subsequently, the abutments were secured using a digital torque gauge (Tohnichi BTG150CN‐S; Tohnichi America), following the torque specifications recommended by the manufacturer (20 N·cm).

Standardized maxillary central incisor cobalt‐chrome alloy crowns (Wirobond 280, BEGO) were milled for each group and cemented on the abutments using a self‐adhesive dual‐curing resin cement (Rely X U200, 3 M Oral Care), as per the manufacturer's instructions.

### Fatigue testing

Three stress profiles for step‐stress accelerated life testing (SSALT), which were derived from the fatigue behavior of narrow implant systems documented in our previous publication [29], were used in this study. Each experimental group comprised 18 specimens (*n* = 18/group), distributed in a 3:2:1 ratio across mild (*n* = 9), moderate (*n* = 6), and aggressive (*n* = 3) stress profiles. These profiles were labeled according to their incremental loading steps, as described elsewhere [20, 30, 33, 34].

**FIGURE 1:**
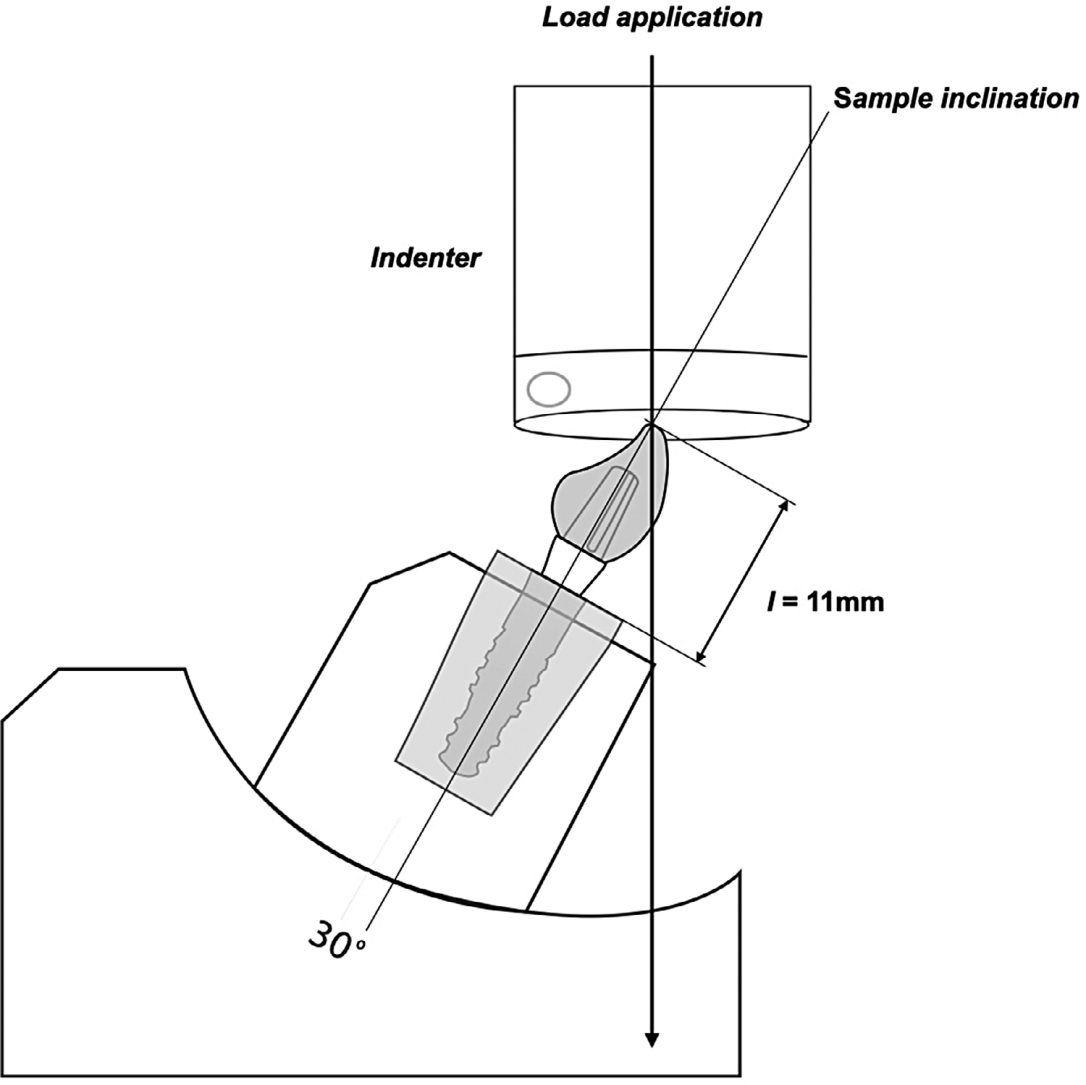
Schematic representation of the experimental set‐up for step‐stress accelerated life testing, where the compression load was applied lingually at the incisal edge of the crown with 30° off‐axis using a flat tungsten‐carbide indenter.

Fatigue testing was performed in an all‐electric dynamic test equipment (ElectroPulsTM E3000 Linear‐Torsion system, Instron) following ISO 14801:2016. The compression load was applied lingually at the incisal edge of the crown with 30° off‐axis using a flat tungsten‐carbide indenter. The moment arm (y) was calculated following ISO 14801 recommendations by the equation y = *l* × sin (30°), where *l* represents the distance between the implant platform and the incisal edge, as illustrated in Figure 1. Based on this calculation, the moment arm was determined to be 5.5 mm. The test was performed at a frequency of 15 Hz until failure (fracture or bending of the abutment, abutment screw, or implant) or survival (completion of profile without failure) until a maximum load of 500 N with samples immersed in distilled water. The load at failure and the number of cycles were recorded according to the stress profiles and the results were analyzed so that a profile of failure behavior could be extrapolated to normal conditions [33].

The data analysis encompassed two main components: a characterization of the life data gathered at different stress levels through an underlying life distribution, and an exploration of the life–stress relationship to quantify how the life distribution changed across these stress levels [33, 35, 36]. The Weibull distribution was selected to model the life data obtained in SSALT. This model facilitated the computation and visualization of the use level probability Weibull curves, which depict the probability of failure versus the number of cycles along with the corresponding 90% two‐sided confidence interval (CI) (Alta Pro 22; Reliasoft). The beta (*β*) value derived from Weibull analysis characterizes the failure rate behavior over time. A *β* value less than 1 (*β* < 1) typically indicates decreasing failure rates over time, often associated with early failures. Conversely, a *β* value greater than 1 (*β* > 1) suggests increasing failure rates over time, which are linked to damage accumulation and fatigue, while a *β* value equal to 1 (*β* = 1) implies failures occurring from a random origin [33]. Since the lower bounds of beta values demonstrated some influence of the material's strength at failure, a Weibull 2‐parameter calculation using the maximum likelihood estimation method was used to determine the Weibull modulus and characteristic strength using the final load to failure or survival. Weibull parameters were then graphically presented in a contour plot where statistical differences are depicted by the absence of contour overlaps.

The reliability, which represents the likelihood of an item to survive a specified number of cycles under a use‐level stress, was calculated for a mission consisting of 100,000 cycles at 80, 150, and 200 N, relevant loads within the physiologic range of maxillary anterior teeth. Group differences were identified by assessing the non‐overlap of the CIs [33, 35, 36].

Failure analyses were conducted in a polarized light stereomicroscope (AxioZoom V16, Zeiss) employing Z‐stack mode, which enables sequential imaging along the *z*‐plane to enhance the depth of focus (ZEN 2.3 PRO, Zeiss).

## RESULTS

**FIGURE 2:**
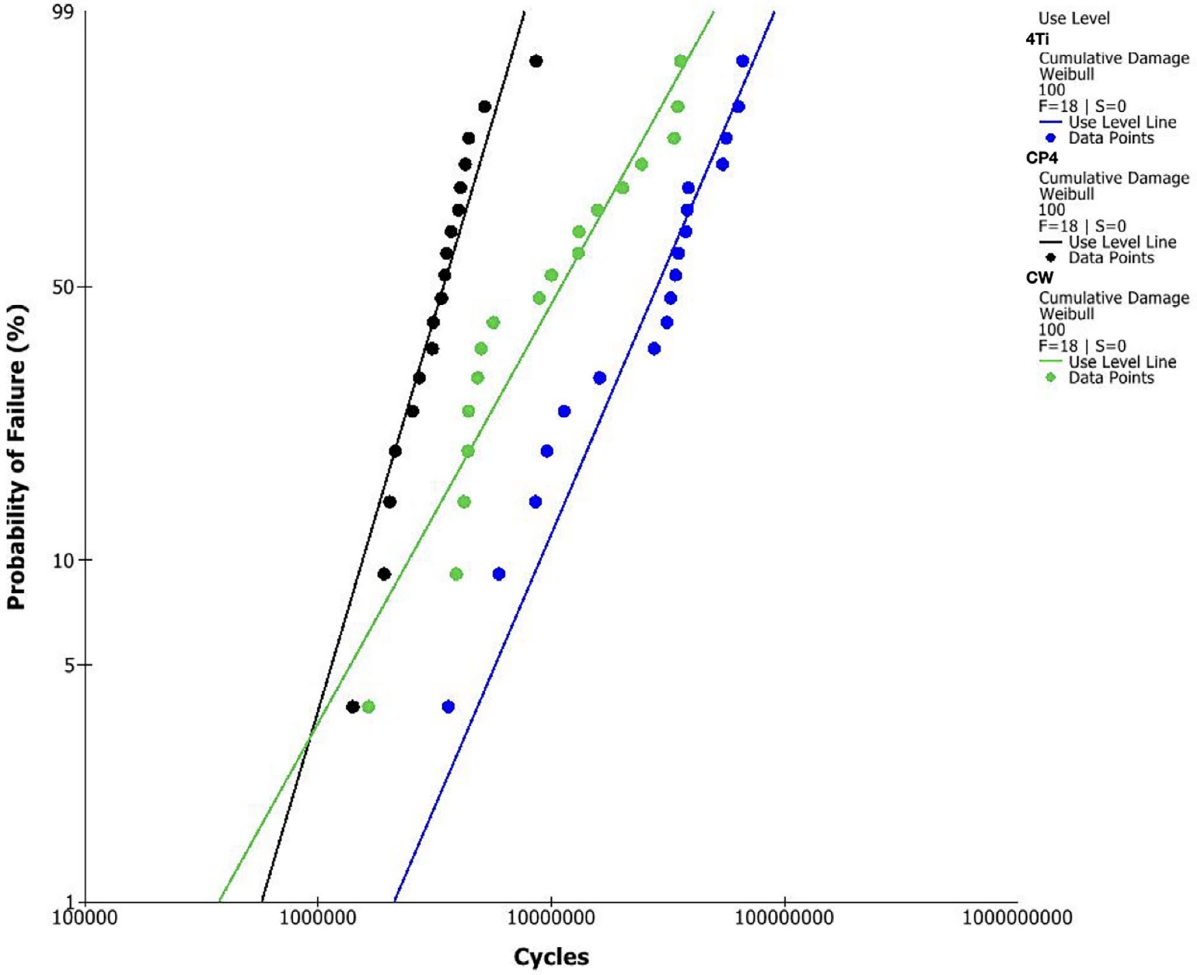
Weibull curves for use level probability of the experimental groups at a set load of 100 N. The probability of failure (%) is depicted as a function of the number of cycles. 4TI, 4Titude titanium bar; CP4, Commercially pure titanium grade IV; CW, Cold worked grade IV titanium.

All specimens failed during SSALT. The resulting beta values for all experimental groups were higher than 1, (*β* = 2.3 [1.2–4.5], 1.2 [0.6–2.3], and 1.6 [0.9–2.6] for CP4, CW, and 4Ti, respectively), which suggests that failures tended to increase over time and were more likely dictated by fatigue damage accumulation. Use‐level probability Weibull curves were calculated from the fatigue testing data for a use‐level load of 100 N and are presented in Figure 2.

The probability of survival of the experimental groups with their corresponding 90% CIs for a mission of 100,000 cycles at 80, 120, 150, and 200 N is presented in Table 1. All narrow implant systems presented high probability of survival for missions at 80, 120, and 150 N (> 99%), regardless of the bulk material used.

**TABLE 1:** Probability of survival with the corresponding 90% CI for a mission of 100,000 cycles and at 80, 120, 150, and 200 N of all experimental groups.

	CP4 Probability (90% CI)	CW Probability (90% CI)	4Titude Probability (90% CI)
Reliability at 80 N	1 (1–1)^A,a^	1 (0.99–1)^A,a^	1 (1–1)^A,a^
Reliability at 120 N	1 (0.99–1)^A,a^	0.99 (0.96–1)^A,a^	1 (0.99–1)^A,a^
Reliability at 150 N	0.99 (0.95–1)^A,a^	0.96 (0.86–0.99)^A,a^	0.99 (0.96–1)^A,a^
Reliability at 200 N	0.45 (0.14–0.72)^B,a^	0.46 (0.18–0.71)^B,a^	0.54 (0.26–0.76)^B,a^

*Note*: Capital superscript letters indicate significant differences between missions, non‐capital superscripts depict significant differences among experimental groups at the same mission.

Abbreviations: 4TI, 4Titude titanium bar; CP4, Commercially pure titanium grade IV; CW, Cold worked grade IV titanium.

For a mission of 100,000 cycles at 200 N, a significant decrease in reliability was observed for all the experimental groups regarding their performance at lower load missions. However, no significant differences in the reliability were observed among different bulk materials.

**FIGURE 3:**
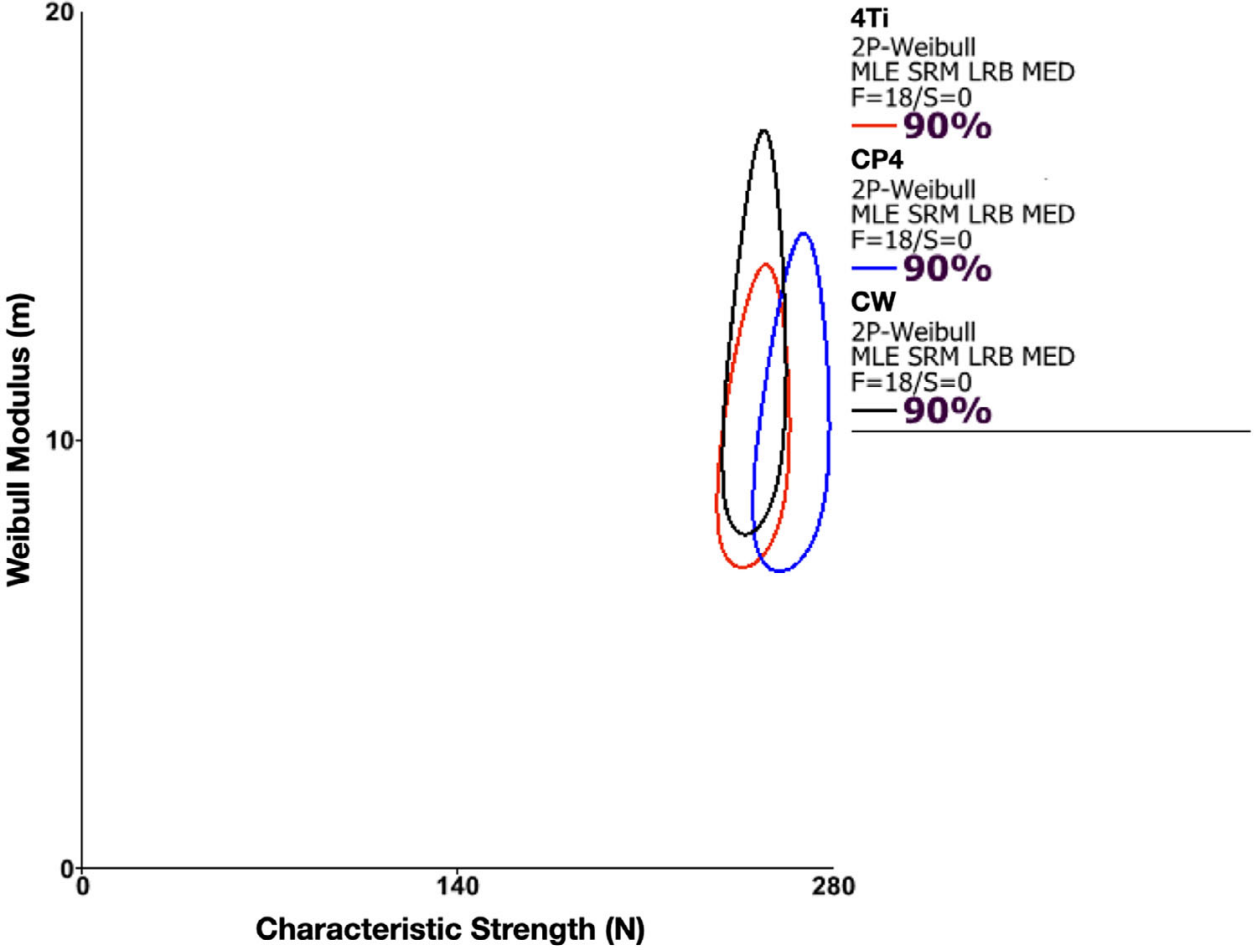
Contour plot showing the Weibull modulus (*m*) as a function of characteristic strength (N). The overlap between contours indicates no statistical difference in Weibull parameters among experimental groups. 4TI, 4Titude titanium bar; CP4, Commercially pure titanium grade IV; CW, Cold worked grade IV titanium.

Considering the lower bound of beta values of CW and 4Ti groups, a Weibull 2‐parameter calculation of the Weibull modulus and characteristic strength using the final load to failure or survival is presented in Table 2. Furthermore, a Weibull modulus versus characteristic strength contour plot was graphed to didactically represent the absence of statistical differences through the overlap of confidence bounds (Figure 3).

**TABLE 2:** Weibull parameters with the corresponding 90% confidence bounds for all experimental groups.

	CP4 Estimate (90% CI)	CW Estimate (90% CI)	4Titude Estimate (90% CI)
Characteristic strength (N)	264 (253–275)^a^	250.5 (241.6–259.3)^a^	250 (239.9–260.3)^a^
Weibull modulus	10.5 (7.7–13.7)^a^	12 (8.7–15.9)^a^	10.4 (7.8, 13.2)^a^

*Note*: Similar superscript letters indicate the absence of significant differences between experimental groups.

Abbreviations: 4TI, 4Titude titanium bar; CP4, Commercially pure titanium grade IV; CW, Cold worked grade IV titanium.

**FIGURE 4:**
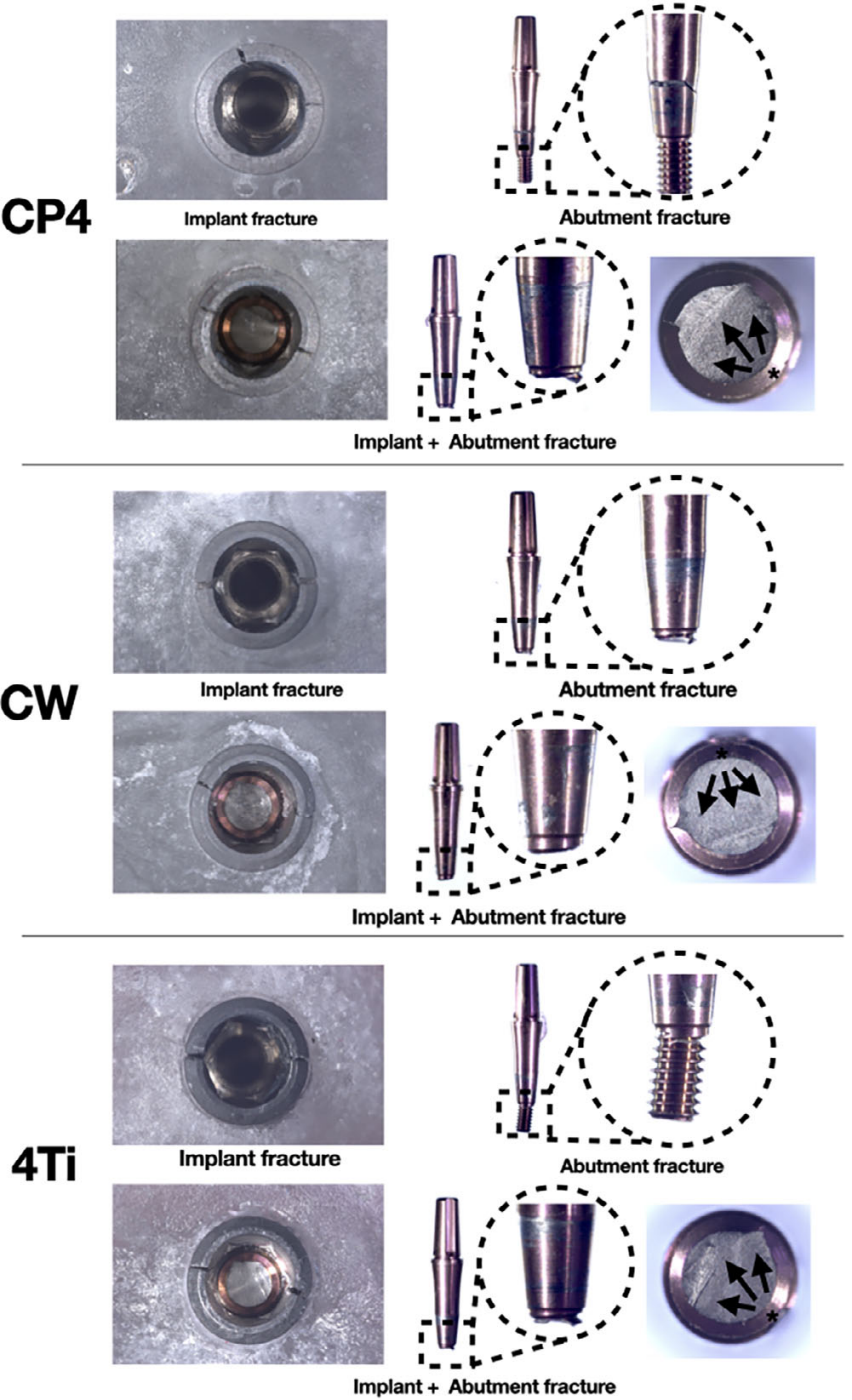
Failure modes of Commercially Pure Titanium Grade IV (CP4), Cold Worked Grade IV Titanium (CW), and 4Titude (4Ti). Failure modes comprised implant fractures, abutment fractures at the lower portion of the conical connection, and combined implant + abutment fractures. Black asterisks and arrows depict the suggested fracture origin and direction of propagation, respectively.

**FIGURE 5:**
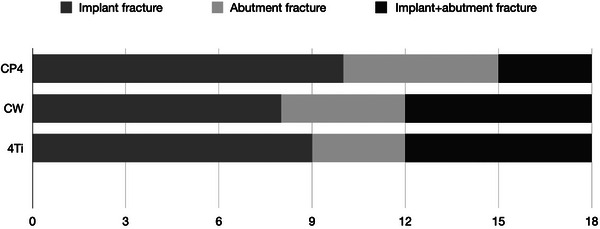
Failure distribution as a function of the narrow implant bulk material. 4Ti, 4Titude titanium bar; CP4, Commercially pure titanium grade IV; CW, Cold worked grade IV titanium.

Similar failure modes were observed for all the experimental groups and comprised implant fracture, abutment fracture, and implant + abutment fracture, as presented in Figure 4. Fractures occurred from the lingual to the buccal aspect, where physiologic forces take place. Furthermore, failure distribution is didactically presented in Figure 5. While similar failure ratios were observed for CW and 4Ti, less implant + abutment fracture were observed for CP4, which demonstrated a slightly higher number of implant fractures.

## DISCUSSION

The development of reduced diameter implant systems based on different bulk materials coupled with the well‐known deleterious effect of repeated physiologic masticatory loading, necessitates a thorough assessment of the mechanical properties of dental implants under conditions that closely mimic cyclic loading in the oral environment. Thus, the current study sought to evaluate the fatigue performance and failure modes of narrow dental implants manufactured with different bulk materials: CP4, CW, and 4Titude bars. The results of the present study demonstrated a high probability of survival for missions under physiologic loads in the anterior region, and no significant differences were observed in the probability of survival or the failure modes of narrow implants regardless of their bulk material. Therefore, the proposed null hypothesis of the present study was accepted.

Data evaluation of fatigue testing provided beta values that suggest increasing failure rates over time, which are linked to damage accumulation and fatigue as the main cause of failure of all tested implant systems. While fatigue predominantly accelerated the failure of the experimental groups, the lower bounds of the CIs also evidenced the influence of the material strength on failure. These findings allowed for a 2‐parameter Weibull evaluation considering the final load at failure, where similar Weibull moduli, unitless parameters that measure the variability of the results, and characteristic strengths, that is, the loads at which 63.2% of the specimens would fail, were observed for all groups. Implants based on CP4, CW, and 4Titude implants exhibited a characteristic strength of 264, 250, and 250 N, respectively, which is above the maximal voluntary force reported for the anterior region (200 N) [37], and similar to the strength previously reported for narrow implant systems [29].

The structural reliability of implant systems is often assessed through the evaluation of Weibull modulus, an indicator of strength variation as a result of flaw population within the material structure. Lower modulus values indicate a non‐uniform distribution of flaw sizes, high data dispersion, and reduced structural reliability [38, 39]. Conversely, higher modulus values are associated with homogeneous flaw size distribution and increased reliability. The narrow implants tested in this study exhibited similar Weibull modulus values regardless of the implant bulk material. The mean values ranged from 7.7 to 15.9, which aligns with previous tests conducted on narrow implants with internal conical monolithic abutments [20].

The probability of survival evaluation for given missions of 100,000 cycles at 80, 120, and 150 N resulted in a high probability of survival for all narrow implants, with no significant differences among bulk materials. Previous in vitro studies that evaluated the reliability of narrow implant systems have reported similar probabilities of survival in missions at 80 and 120 N for cold‐worked grade IV and titanium–zirconium alloys [29, 31]. Therefore, narrow implants manufactured with the bulk materials tested in the present study may be considered a reliable alternative for the rehabilitation of lost or doomed anterior teeth, since the mean physiologic masticatory forces in these regions vary within the range from 25 to 45 N [40]. Considering critical scenarios of loading and the maximum voluntary force peaks reported in the anterior region [37], the reliability of narrow implants for missions at 200 N was also evaluated. As expected, a decrease of approximately 40% to 50% in the probability of survival was observed for all groups, with no significant differences among bulk materials. Therefore, caution is recommended when considering the use of narrow implants in areas subjected to higher mechanical loading, such as canines and posterior regions of the mouth, where narrow implants may be prone to fracture and mechanical complications [19, 20, 30].

From a mechanical perspective, the development of cold working processing and titanium alloys has proven its superiority regarding commercially pure titanium [23, 41]. Titanium aluminum vanadium, Ti6Al4V, or Grade 5 titanium alloy has been used by some manufacturers due to its highlighted mechanical properties regarding CPT grade IV [22]. However, negative effects on cell viability have been associated with the release of aluminum and vanadium, with a consequent adverse influence on implant biocompatibility [42]. Therefore, cold working grade IV CPT has been suggested as an alternative to improve the mechanical properties of CP4 without the cytotoxic concerns associated with Ti6Al4V [43]. Cold working is the method of strengthening metal by altering its shape without employing heat, where mechanical stress is applied to metals, resulting in a permanent alteration of their crystalline structure that leads to enhanced strength [43]. Furthermore, 4Titude titanium bars produced by severe plastic deformation are reported by the manufacturer to present a nominal tensile strength of 1172 MPa, which is significantly superior to CP4 and similar to the strength reported for cold‐worked CP4. While the mechanical properties reported by the manufacturer are promising, little has been reported in the scientific literature regarding the processing method of 4Titude titanium bars, which warrants further investigation. Despite the significant differences in mechanical properties reported for the bulk materials utilized in this study [25], our findings depicted no significant differences in the characteristic strength and reliability evaluations of narrow implants as a function of the bulk materials. This might be due to the reduced thickness of the implant walls, which may be the critical factor dictating failure through fatigue damage accumulation by repetitive loading, rather than to the strength of the bulk material itself [44].

Regardless of bulk material, the narrow implants tested in the present study presented similar failure modes comprising implant or abutment fracture, or the combination of both. These findings differ from those reported in the previous literature [29], where the failure mode was chiefly found to comprise abutment and screw fractures while no implant fractures were reported. Such a difference might be explained by the different abutment selection for each study. While Ti‐base abutments were used by Bergamo et al. [29], monolithic abutments for cemented restorations were used in the present investigation, which present a bulkier structure than two‐piece abutments. Although internal conical connections have been suggested to improve the stability and stress distribution of implant reconstructions as a function of a deeper engagement on the implant–abutment interface, the use of monolithic abutments in narrow diameter implants resulted in unfavorable scenarios for the thin implant walls. The effect of damage accumulation and progressive crack growth at the weakest component of the implant‐supported reconstruction resulted in competing failure modes between the thin implant wall and the solid abutment.

From a biological standpoint, failure modes involving implant fractures often lead to higher morbidity scenarios compared with abutment failures. The removal of fractured implants, aside from being technically challenging, typically involves some compromise of surrounding tissues [45]. Moreover, failure modes involving abutment fractures are often attributed to fatigue damage accumulation at the thinnest portion of solid abutments with fractures occurring at the top of the screw threads, as observed in this study. This scenario presents an opportunity for the removal of fractured screws and the replacement of prostheses, with fewer biological and financial expenses.

Although the present study did not strictly adhere to the ISO 14,801, specifically by not using a hemispherical loading cap and not simulating 3 mm of bone resorption, these modifications were intended to better replicate clinical conditions. The use of anatomically representative crowns and the preservation of bone‐level placement reflect more common scenarios in anterior implant restorations, particularly in subcrestal installations where crestal bone stability is expected [46, 47, 48]. While ISO 14,801 provides a standardized worst‐case framework for comparative testing, our approach aimed to evaluate the fatigue performance and failure modes of narrow‐diameter implants under loading conditions that more closely approximate physiologic masticatory forces and implant geometry in vivo. This design choice enhances the translational relevance of the findings, supporting their application to clinical situations where implant success is influenced by both mechanical and anatomical factors.

The step‐stress accelerated life testing methodology employed in this study has been extensively utilized to assess the mechanical behavior of dental implants and their associated components, enabling the timely extrapolation of clinical failure patterns [20, 21, 33, 34, 49, 50]. While high reliability and characteristic strength were observed for narrow implants at physiologic loads for the anterior region in the present study, further clinical evaluations are warranted to detect whether the long‐term survival of narrow implant‐supported reconstructions is affected by implant's bulk material.

In conclusion, narrow implants presented a high probability of survival for physiologic masticatory forces in the anterior region. Characteristic strength, reliability, and failure modes were similar regardless of the bulk material, which suggests that fatigue damage accumulation at thin implant walls dictated failure over bulk material strength.

## AUTHOR CONTRIBUTIONS

**Conceptualization**: Estevam A. Bonfante, Paulo G. Coelho, and Ernesto B. Benalcázar‐Jalkh. **Formal analysis**: Ernesto B. Benalcázar‐Jalkh, Edmara T. P. Bergamo, and Lukasz Witek. **Investigation**: Ernesto B. Benalcázar‐Jalkh, Edmara T. P. Bergamo, Adolfo C. O. Lopes, Laura F. de Carvalho, and Abbas Zahoui. **Methodology**: Estevam A. Bonfante, Paulo G. Coelho, Lukasz Witek, and Ernesto B. Benalcázar‐Jalkh. **Software**: Ernesto B. Benalcázar‐Jalkh, Edmara T. P. Bergamo, and Laura F. de Carvalho. **Validation**: Adolfo C. O. Lopes, Edmara T. P. Bergamo, Paulo G. Coelho, Lukasz Witek, and Laura F. de Carvalho. **Visualization**: Adolfo C. O. Lopes and Abbas Zahoui. **Funding acquisition**: Estevam A. Bonfante. **Supervision**: Estevam A. Bonfante. **Writing—original draft**: Ernesto B. Benalcázar‐Jalkh and Abbas Zahoui. **Writing—review and editing**: Estevam A. Bonfante, Paulo G. Coelho, Lukasz Witek, Edmara T. P. Bergamo, Adolfo C. O. Lopes, Laura F. de Carvalho, and Abbas Zahoui.

## CONFLICT OF INTEREST STATEMENT

The authors declare that there is no conflict of interest regarding the publication of this paper.

## Data Availability

Data will be available upon request.
